# The Aetiology, Histopathology, and Ultrastructural Features of Perianal Erythema (Red Anus Syndrome) in the European Eel (*Anguilla anguilla*)

**DOI:** 10.1371/journal.pone.0090790

**Published:** 2014-03-07

**Authors:** Omar A. S. Tamam

**Affiliations:** Department of Natural Resources, Environmental Studies & Research Institute, University of Sadat City, Sadat City Campus, Egypt; National Science and Technology Development Agency, Thailand

## Abstract

The European eel (*Anguilla anguilla*) is a critically endangered species. Red anus syndrome (RAS) is known to be associated with parasitic infections of the eel, particularly with *Anguillicola crassus*, but the full range of causative pathogenic organisms has not been systematically investigated. Here we examined the infective organisms and histopathological and ultrastructural features of seventy eels with RAS. In total, nine different pathogens were detected in association with RAS: *Pseudomonas aeruginosa* were present in twelve specimens (17%), the metacercaria of *Euclinostromum heterostomum* in three cases (4%), *Gastrostome* (Bucephalidae family) in seven cases (10%), *A. crassus* in forty-five cases (64%), *Bothriocephalus* in seventeen cases (24%), and *Proteocephalus* in twenty-three cases (32%). Yeast, amoeba, and myxobolus-like pathogens were seen in the anal skin in all cases when examined in combination with electron microscopy. Histopathologically, the lesions appeared as anoproctitis of varying severity from mild anusitis to severe haemorrhagic anoproctitis, with severe perianal oedema, haemorrhage, and proctoptosis. Gut inflammation ranged from mild catarrhal enteritis to severe haemorrhagic enteritis with mucosal sloughing. RAS is associated with a range of parasitic infections, not only *A. crassus*, some of which we describe here for the first time. Since RAS is not associated with direct invasion by parasites, it is likely that RAS is a secondary phenomenon caused by superadded infection on a background of generalised immunosuppression, or indirect local toxic effects. RAS may be used as a non-invasive indicator of underlying parasitic infection, but further investigations are required to establish the causative organisms for effective fishery management.

## Introduction

The European eel, *Anguilla anguilla*, is common to the waters of Europe, Asia, and the Mediterranean basin. The European eel has undergone a dramatic decline in population since the 1970s due to overfishing, pollution, habitat loss, and susceptibility to infection [1]; consequently, the European eel is a critically endangered species. The eels remain an important source of economic value to many communities and they are an important source of food. Therefore, understanding the factors that might influence or be useful to sustainable fishing practices remains an important area of study.

Perianal erythema, also termed the ‘red anus syndrome’ (RAS), is a recognised external morphological manifestation of eel pathology, in particular infection with parasites [2]. Perianal erythema is best described in association with infection with the swimbladder nematode *Anguillicolla crassus*, which causes retarded growth, increased susceptibility to other disease, and, in severe cases, death of the fish [3]. In one study, *A. crassus* was present in as many as 80% of eels with perianal discoloration, and therefore this feature alone may be a useful non-diagnostic tool for use in fisheries management [2].

However, perianal erythema has also been described in association with other eel infections, such as the nematode *Camallanus lacustris*, and therefore the feature is unlikely to be specific for one particular infection [4]. We therefore sought to undertake a systematic analysis of the organisms associated with RAS in a large number of European eels with anal discoloration from the Domiat Coast region of Egypt. In addition, we comprehensively examined the histopathological and ultrastructural features of the gastrointestinal tracts of eels with RAS, in order to better understand the pathogenesis of this collection of diseases. Together, these analyses provide a valuable contribution to understanding the causes and effects of eel pathologies, so that fisheries can better formulate management strategies for sustainable aquaculture.

## Methods

### Ethics statement

Although *A. anguilla* is a critically endangered species, there remains widespread legal fishing activity for human food consumption in Egypt and worldwide; hence the need to conduct this study to guide effective fishery management and help restore stocks. The eels were obtained from fish market on their way for human consumption as part of the legal eel fishing industry in Egypt. The eels were euthanized by decapitation by fishermen working under the regulated authority of the General Authority for Fish Resources Development, Egypt. Since the study did not use laboratory animals, was performed as part of the investigation of natural disease, and used animals already in the food chain, no ethical approval from an animal ethics committee was required.

### Fish samples and histopathology

**Figure 1:**
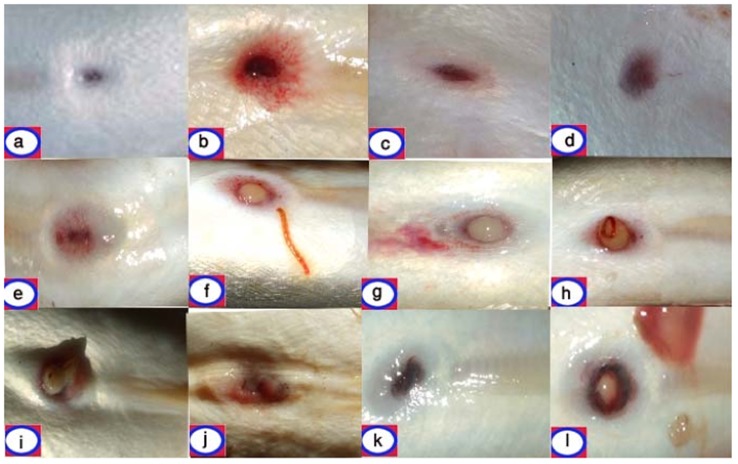
The appearances of the eel anal opening. Normal (**A**), different degrees of perianal erythema (**B**–**G**) with the presence of a blood worm (Chironomid midge larva) on the skin of the eel in sample (**F**). Perianal oedema and proctoptosis (**H**–**J**) and perianal black pigmentation (**K, L**).

Seventy live European eels with RAS were obtained from fish market between March 2010 and December 2011; 0–3 fish were examined during any one period. Eels originated from the Domiat region of the Nile Delta in Egypt. Only fish with visible perianal erythema were selected for examination (see Figure 1). The selected fish were characterized by the presence of either red, reddish brown, or grey zones around the anal opening; in addition, anal prolapse was apparent in some eels.

Each specimen underwent a full postmortem examination, as described previously [5]. In particular, the intestine was opened longitudinally for detailed examination. Samples from the anal opening, rectum, large intestine, small intestine, and swim bladder were immediately fixed in 10% phosphate-buffered formalin. Fixed tissues were processed using standard fish histology protocols, sectioned at 5 µm, and stained with haematoxylin and eosin and periodic acid Schiff (PAS) for subsequent microscopic analysis and photomicrography using a digital camera [6].

### Scanning electron microscopy (SEM)

Three 0.5 cm thick fresh tissue samples were taken from the anal opening to include the rectal mucosa and placed in 5% cold-buffered glutaraldehyde for two days. Samples were then washed in cacodylated buffer for thirteen minutes three times, post-fixed in 1% osmium tetroxide for two hours, and then dehydrated in a graded ethanol series (30%, 50%, 70%, and 90%) over a period of two hours. Samples were left in 100% ethanol for two days, and then amyl acetate for a further two days. Finally, liquid carbon dioxide was used to achieve critical-point drying, samples were stuck to metallic blocks using silver paint in a sputtering apparatus, and samples were evenly coated with gold (15 nm). SEM was performed using the JEOL JSM 5400 LV scanning electron microscope at 15 kV.

### Microbiological examination

Swabs were obtained from the anal opening using aseptic technique and were cultured in nutrient broth for one day then culture on blood agar in order to culture and detect pathogenic bacteria. Colonies were picked and re-streaked onto new plates of *Pseudomonas* isolation agar as described in [7]. The bacterial isolates were identified according to the biochemical reaction scheme provided by Finegold and Martin [8]. Fungal organisms were assessed using light microscopy.

### Parasitological examination

The intestine was opened longitudinally and its contents examined for parasites using a digital stereomicroscope. Live parasites were collected from the intestines of infected eels and fixed and processed using standard methods [9], [10]. Identification was made on whole-mount sections according to the criteria shown in Table 1.

**Table 1:** Criteria used for the identification of the main parasites seen in the eels.

Parasite	Features used to identify organism
***Euclinostomum heterostomum***	Whole-mount of *Euclinostomum heterostomum* metacercaria identified morphologically according to the morphology of the acetabulum, anterior testis, cecal diverticulum, cirrus pouch, intestinal cecum, ootype, ovary, posterior testis, uterus, and uterine sac. The whole-mount specimen was readily identified as *E. heterostomum* according to previous descriptions of this species [23]. The larvae measured 5 mm long by 2 mm wide, with measurements of other structures falling into the ranges given for this species (see [23]).
***Gastrostome***	Bucephalidae is a large family of digenean trematodes that have no suckers, but a muscular attachment organ at its anterior end termed a “rhynchus” [24]. *Gastrostome* belongs to this family.
***Bothriocephalus***	This genus has a characteristic scolex that is characterized as elongated, somewhat depressed, with a bilobedapical disk whose bothrial edges are indented, the two indentations being connected by a groove [25].
***Proteocephalus***	The presence of four suckers on the scolex is common for this genus. The reproductive system is similar to the scheme of the *Cyclophyllidea*. Adult worms are usually 10–30 cm long. At the posterior margin of the proglottid is a dark red, bilobed ovary. The lateral bands or dark spots are the vitellaria. On the margins of the proglottids, alternating irregularly, are the genital pores, with a prominent cirrus pouch proceeding medially from the genital pore. Almost filling the centre of each proglottid is the uterus (its lobed margin can be seen clearly in the topmost complete proglottid), filled with small, dark red eggs. In some areas where the eggs have not obscured them, the testes can be seen as larger, less-intensely-staining circles [26].
***Myxobolus***	Myxospores, which develop from sporogonic cell stages inside fish hosts, are lenticular. They have a diameter of about 10 micrometers and are made of six cells. Two of these cells form polar capsules, two merge to form a binucleate sporoplasm, and two form protective valves [27].

## Results

### Gross external examination and diagnosis of red anus syndrome

The normal eel anus is identified as a small, punctate depression on the underside of the fish. A healthy specimen will show no signs of erythema or swelling (Figure 1A), with anal colour the same as the lateral surface skin colour. In fish with red anus syndrome, a range of appearances is apparent, depending on severity. In mild lesions there were telangiectatic or punctate erythematous changes confined to the edge of the anal orifice (Figure 1 B–G). In more advanced cases, there was marked perianal oedema, haemorrhage, and proctoptosis (Figure 1 H–J). Chronic lesions were identified by brown-black discoloration and the anal tissue acquired a hard texture with irregular, but well defined, borders (Figure 1 (K, L). Although no ectoparasites were observed, occasional blood worms (chironomid midge larvae) were present and adherent to the eel skin as non-pathogenic water contaminants (Figure 1F).

### Parasites and bacteria associated with red anus syndrome

*Pseudomonas aeruginosa* organisms were isolated from the anal smears of twelve specimens (17%). This pathogen was always associated with severe anoproctitis. No other pathogenic bacteria were detected.

**Figure 2:**
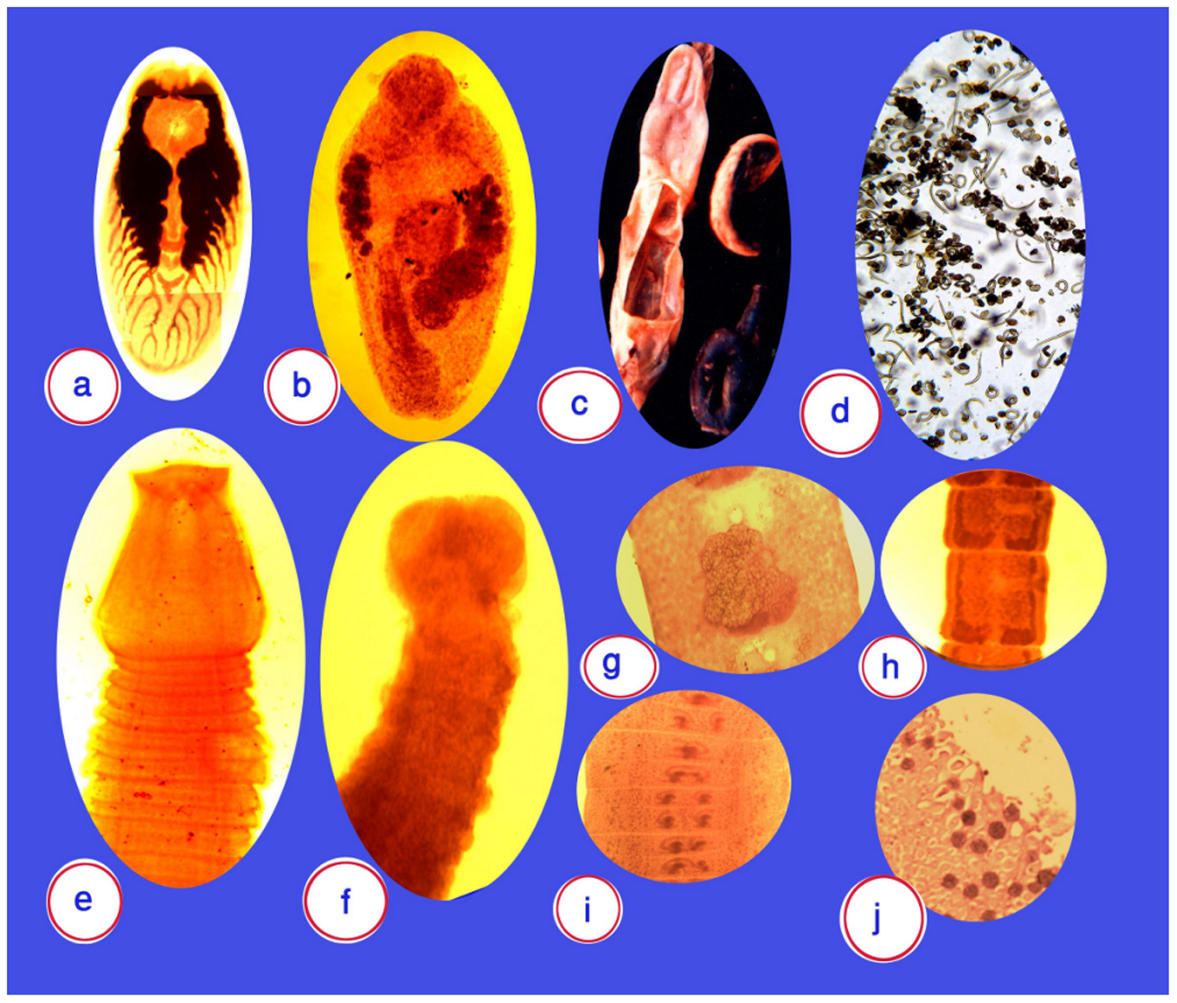
The parasites found in the eels. (**A**) Metacercaria of *Euclinostromum heterostomum,* (**B)** *Gastrostome,* a member of Bucephalidae, (**C**) Dissected swimbladder and *Anguillicola crassus,* (**D**) Swimbladder fluid containing numerous *Anguillicola crassus* larvae, (**E**) *Bothriocephalus* scolex, (**F**) Proteocephalus scolex, (**G**) *Bothriocephalus gravid* segment, (**H**) *Proteocephalus* mature segment, (**I**) *Bothriocephalus* mature segment, (**J**) Myxobolus-like cysts invading the skin.

In total, five different metazoal parasite species were detected. The metacercariae of *Euclinostomum heterostomum* were detected in the intestinal lumen in three cases (4%; Figure 2A); *Gastrostome,* a member of the Bucephalidae family, in seven cases (10%; Figure 2B); *A. crassus* in 45 cases (64%; Figure 2 C, D); *Bothriocephalus* in 17 cases (24%; Figure 2 E, G, I); and *Proteocephalus* in 23 cases (32%; Figure 2 F and H). Yeast, amoeba, and myxobolus-like pathogens were noted in all cases. Myxosporean-like parasite cysts were recorded in all cases, which mainly invaded the perianal subcutaneous tissues (Figure 2J).

**Figure 3:**
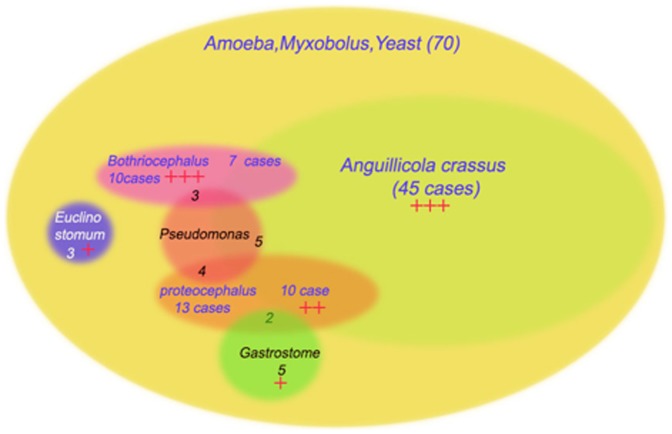
Red anus syndrome is complex and multifactorial. This diagram shows the distribution of the different infective organisms, and highlights that infection with 3 or more organisms is not uncommon. The intensity of parasitic infection in the fish is indicated by + <3, ++ 3–10, +++ >10 parasites per host.

Mixed infections were common, as can be seen in Figure 3. In all cases, amoeba, myxobolus-like organisms, and yeast were detected using the combination of techniques. *A. crassus* was associated with other infections in nearly 50% (22/45) of cases; *Bothriocephalus* were found in seven cases, *Pseudomonas aeruginosa* in five cases, and *Proteocephalus* in ten cases. *Proteocephalus* and *Gastrostome* infections were present together (as co-infections) in two eels, and *Euclinostomum*, in itself a rare disease, always occurred in isolation (i.e., with no other concurrent infection).

### Histopathologic features of red anus syndrome

**Figure 4:**
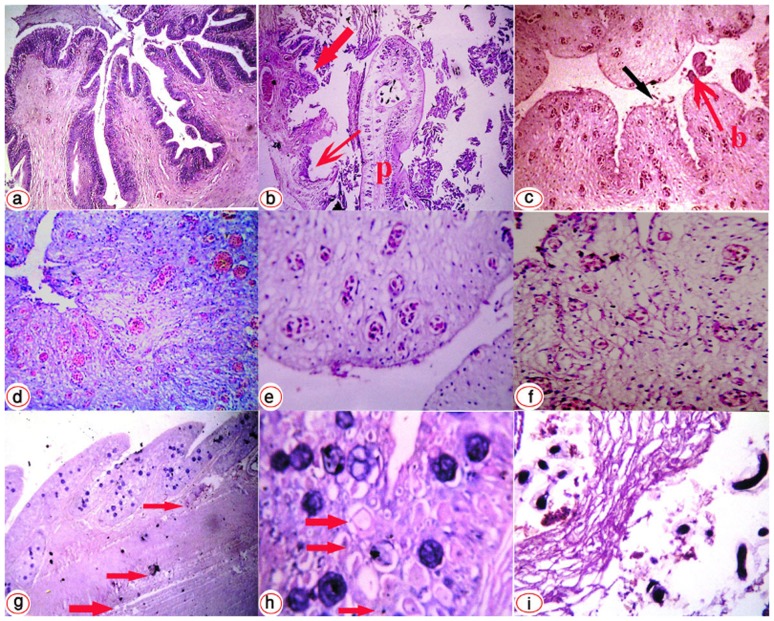
Photomicrographs of the intestine of an eel. (**A**) normal (H&E x10). (**B**) Cross section of eel intestine with specimens of *Protocephalus cestode* free in the lumen (P). An apical erosion of a villus and complete sloughing of the intestinal mucosa can be seen (arrows), with severe degeneration and necrotic changes in the mucosa and submucosa (H&E x40). (**C, D**) Cross section of eel anal opening showing sloughing of the anal epithelium (arrow) with bleeding and congested vessels (H&E x20). (**E**) Submucosa shows dilated congested capillaries (H&E x40). (**F**) Severe oedema separating the connective tissue fibres, inflammatory infiltrate, and red blood cells in the anal mucosa (x40) (**G**) Longitudinal section through the anal opening, with small millet-sized nodules containing a large number of *Myxobolus* species (H&E x10), and arrows indicating focal necrosis. (**H**) Anal skin showing severe degenerative and necrotic changes, mucus, and club cells (arrows) of the epidermis with oedema and an inflammatory cell infiltrate (H&E x40). (**I**) Tissue rupture and degeneration of the swimbladder, containing numerous larvae. The larvae are surrounded by cell debris from the necrotic submucosa (H&E x10).

In the small intestine, mild catarrhal enteritis was observed in fish with trematode infection; this may have been due to the trematodes themselves or one of the co-infections. Small intestinal villi were covered with mucus, inflammatory cells, and epithelial cell debris from excessive sloughing. In fish with cestodal infection, there was severe haemorrhagic enteritis, with sloughing of the mucosal epithelium, severe congestion of submucosal blood vessels, and a protein-rich exudate containing active chronic inflammatory cells including macrophages, lymphocytes, and eosinophil granulocytes the surface (Figure 4B). Necrotic changes involved the mucosa and extended into the submucosa.

In the large intestine in the majority of cases, but in particular *Proteocephalus* infestation with *Pseudomonas* infection, demonstrated massive epithelial and mucosal necrosis, submucosal oedema, widespread haemorrhage, and an associated active chronic inflammatory cell infiltrate.

At the anal opening there was clear evidence of anusitis. There was sloughing of the anal canal epithelium with rupture of superficial capillaries (Figure 4C and D). Submucosal capillaries were ectatic and congested (Figure 4E), and the lamina propria was oedematous with extravasation of red blood cells and separation of the connective tissue fibres (Figure 4F). Myxobolus-like cysts were seen invading the perianal dermis to cause focal necrosis (Figure 4G), with the skin itself showing severe degenerative and necrotic changes in the epithelium with ‘club cells’ and an associated inflammatory exudate (Figure 4H).

The changes in the swimbladder were only seen with anguillicolosis, as expected and previously described [11]. There was widespread extravasation of red blood cells. In some cases, there was also evidence of old haemorrhage in the form of hemosiderin deposition, and eosinophils were occasionally seen. Due to the presence of exudate, the muscle layer was loose with separated the muscle fibres and dilated capillaries. The submucosa of the swim bladder contained numerous larvae, which were surrounded by cell debris from the necrotic and degenerate submucosa (Figure 4I).

### Electron microscopic features of red anus syndrome

**Figure 5:**
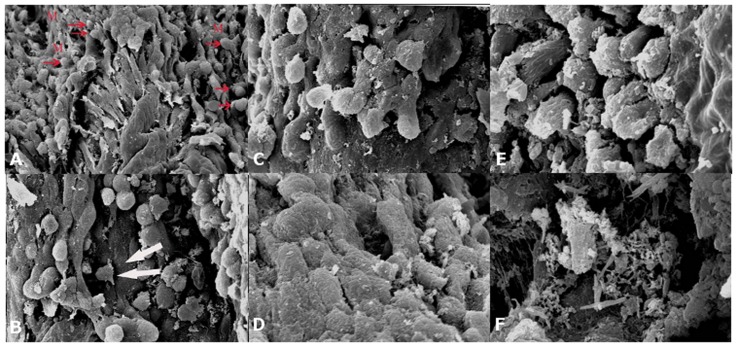
Scanning electron micrographs of the lower intestine of an eel with anal redness. Enterocytes are not clearly visible due to mucus coating (M). (**A**) Spherical yeast cells and cocci (arrow) captured in the mucus covering the intestinal epithelium. (**B**) Typical view of mixed infection by yeast, bacteria, and amoebae with characteristic pseudopodia and membrane blebbing (arrow) (**C**) Close association of yeast cells and intestinal mucosa. (**D**) Derangement of the enterocytes, especially on the right as the cell borders were not clearly defined with epithelial loss. (**E**) The surface is partially covered with mucus and a fibrinoid pseudomembrane. (**F**) The enterocytes are covered with mucus-fibrinoid pseudomembrane, which after dehydration appear as fibrous strands of material.

A wide spectrum of anorectal mucosal abnormalities were observed using SEM, as shown in Figure 5A–F, and the technique was particularly useful for revealing the 3-dimensional view of the surface environment. In areas without observable lesions, the mucosa was not clearly visible when viewed at low magnification due to an intact mucus layer (Figure 5A). However, at higher magnification, spherical yeast cells and cocci were clearly visible in the mucus covering the intestinal epithelium. In areas with visible abnormalities, the mucosal organization was altered due to the presence of mixed infection with yeast, bacteria, and amoeba demonstrating characteristic pseudopodia and membrane blebbing (Figure 5B). Yeast cells were intimately associated with the intestinal mucosa (Figure 5C). The glandular borders were oedematous, and the gland lumen was dilated due to epithelial loss (Figure 5D). In addition, the enterocytes were covered with a protein-rich pseudomembrane containing material composed of a network of filaments with a fibrin-like structure (Figure 5E). When condensed and dehydrated, this material was most apparent as fibrous strands (Figure 5F).

## Discussion

This study is the first comprehensive analysis of the aetiology, histopathology, and ultrastructural features of European eels with RAS. As expected, *A. crassus* was frequently observed, followed by *Proteocephalus (32%), Bothriocephalus (24%), Gastrostome (10%), and Euclinostomum heterostomum (*4%). All eels were infected with more than one organism, confirming that RAS is a manifestation of a number of different infections that frequently co-exist.

Although RAS has previously been described in association with *A. crassus*, other organisms have also been implicated in the pathogenesis. The nematode *Camallanus lacustris* has also specifically been described in association with RAS [4], while others have described the phenomenon in more general terms; Van Banning and Haenen attributed RAS to bacterial infection [12], while Rodjunk and Shelenkova reported that RAS is secondary to parasite–induced enteritis, without naming specific organisms [13]. Here, for the first time, we describe RAS in association with *Euclinostomum heterostomum, Gastrostome,* and *Bothriocephalus*. Other parasites, such as *Myxdium spp., Trichodina spp, Dactylogyrus pseudodactylogyrus, Anguillicola cruses, and Pseudoergostla* have all been described in European eel in Egyptian waters, but these were not observed in association with RAS in this study [11]. Most strikingly, nearly 50% of eels with *A. crassus* infection also harboured another specific infection, demonstrating the complex and multifactorial aetiology of RAS. RAS is certainly not specific for *A. crassus*, and fisheries need to perform more specific tests if the causative organisms need to be established for management decisions.

In addition to the metazoal species identified, 17% of cases were shown to harbour *P. aeruginosa* by microbiologic culture; no other bacterial species were identified on routine culture. Gram-negative infections, including *Pseudomonas*, are not uncommon in warmwater fish and in order to successfully colonise and be pathogenic must overcome host defences, as appears to have been the case here [14], [15]. *Myxobolus* species have been described in the European eel since the early 1900s and are a common infection of the fish over a wide range of geographic locations from Europe to Japan, but poor morphologic and taxonomic characterization makes subclassification difficult; hence the term ‘myxobolus-like’ is used in this paper [16], [17]. Although the specific types of amoebae and yeast were not formally identified in this study, various types of amoebae are common secondary infections in fish [4] and fungal infections, including with yeasts, have been reported to be pathogenic in eels in fish farms [18]. However, to my knowledge this is the first description of these organisms in association with RAS.

The prevalence of *A. crassus* (64%) was entirely consistent with the previously described association with RAS [2], and at the high end of the prevalence reported for the general eel population (from 11% to 80%), as would be expected from a population enriched in disease [19], [20]. *A. crassus* causes damage to the swimbladder by sucking blood from its wall, causing direct damage to the mucosa as seen in this study [21], [22]. The mechanism of perianal erythema, which is distant from the primary site of infection, is less well understood. It has been suggested that the red and swollen anus is due the release of eggs and larvae to the intestine via the pneumatic duct, which then induce severe inflammation, or due to bacterial infections in the posterior region of the abdomen [12]. Our SEM data suggest that the perianal erythema is not directly associated with the presence of eggs or larvae of *A. crassus*, which were not present in the perianal skin, but is due to superadded infection with amoeba, fungi, and myxobolus-like organisms, which were evident in every case. It therefore appears that the perianal erythema induced by *A. crassus* is a secondary phenomenon, either due to generalised immune compromise, or local mucosal weakness cause by luminal toxins or organisms.

Although the parasitic infections affecting the European eel have been extensively studied, the histopathological features of the damage they cause are less well described. Abdelnomen et al. recently presented a comprehensive overview of the distribution and histological features of European eel infected with parasites, and described similar features of *A. crassus* infection of acute on chronic inflammation of the swimbladder with erosion and ulceration in the most severe cases [11], [22]. Likewise, the other RAS-associated organism detected in their study, *Proteocephalus spp.*, showed features of mild enteritis. Even when infected with the same organism, European eels show a spectrum of pathological abnormalities; for instance, in one study, infections with *A. crassus* resulted in pathological abnormalities in the swimbladder in only 28% of infected cases [19]. Since all eels with RAS and *A. crassus* had visible pathological abnormalities in this study, it may be that RAS only becomes associated with underlying parasitic infection in the most severe cases.

## Conclusions

Red anus syndrome commonly affects European eels. Far from only being associated with *A. crassus* infection, RAS is associated with a range of infections, some of which we describe here for the first time. Parasitic infections cause a range of severity of enteritis, but most strikingly the ultrastructural studies reveal that perianal erythema is always associated with superadded fungal, bacterial, and amoebic infections. It is therefore likely that RAS is a secondary phenomenon caused either by generalised immunosuppression, or indirect local toxic effects caused by the presence of parasites that allow superadded infection to occur. RAS may be used as a non-invasive indicator of underlying parasitic infection, but further investigations are required to establish the causative organisms for effective fishery management.
